# Supplementary material for: Comparative Histology of C Thyrocytes in Four Domestic Animal Species: Dog, Pig, Horse, and Cattle
Source: Animals (Basel). 2022 May 23;12(10):1324. doi: 10.3390/ani12101324 (PMC9137929; doi:10.3390/ani12101324)
Supplement: Supplementary file 1 [file animals-12-01324-s001.zip › animals-1703632-supplementary.pdf]

Supplementary Table S1. Fraction of C thyrocytes in three different regions of the medial part of canine thyroid lobe and total fraction of C thyrocytes in examined cases.

Case no.	Fraction of C thyrocytes (%)			Total fraction of C thyrocytes per case (%)
	Superficial area	Central area	Intermediate area	
	Mean±SD (Range of values)	Mean±SD (Range of values)	Mean±SD (Range of values)	Mean±SD
1	1.61±3.22 (0-8.21)	18.71±13.51 (0-44.75)	12.68±10.39[c] (0-35.87)	13.15±12.12
2	1.31±2.85 (0-10.26)	11.18±9.34 (0-25)	6.09±7.11[b] (0-21.58)	7.17±7.93
3	2.32±4.69 (0-14.03)	11.57±6.29 (0-22.81)	5.16±5.41[c] (0-15.93)	7.04±6.64
4	2.03±4.76 (0-17.08)	8.19±4.43 (0-22.18)	5.70±7.15[b] (0-22.18)	6±6.02
5	1.8±2.76 (0-7.59)	11.58±5.98 (0-23.65)	6.07±4.96[c] (0-15.35)	7.63±6.16
6	0.65±2.18 (0-8.2)	8.26±7.13 (0-22.52)	5.84±6.44[b] (0-14.18)	5.48±6.41
7	1.77±5.42 (0-14.91)	6.67±5.4 (0-13.79)	5.56±6.17[b] (0-19.2)	5.36±5.42
8	4.02±8.86 (0-31.64)	17.73±11.79 (0-33.16)	13.87±12.6[b] (0-40.27)	14.28±12.39
9	4.27±6.33 (0-19.28)	19.71±10.61 (0-34.87)	13.34±15.39[b] (0-44.21)	14.44±12.83
10	2.87±5.13 (0-15.24)	17.57±6 (5.55-23.98)	10.72±7.67[c] (3.24-30.34)	10.4±8.68
11	3.9±542 (0-16.23)	16.64±9.8 (0-35.18)	11.87±10.62[b] (0-33.15)	11.31±10.19
12	0.77±1.93 (0-6.9)	14.68±12.47 (1.82-43.48)	6.85±5.89[c] (0-16.78)	7.78±9.73
13	1.95±4.39 (0-16.11)	11.04±6.27 (3.12-25.87)	5.17±5.92[c] (0-18.63)	6.63±6.65
14	1.62±3.1 (0-10.13)	7.4±5.74 (0-17.51)	6.88±5.99[b] (0-16.72)	5.87±5.65
15	0.67±1.78 (0-6.67)	5.45±5.83 (0-19.34)	4.68±5.19[b] (0-15.42)	3.97±4.98
16	1.14±1.58 (0-4.69)	9.89±5.83 (0-19.11)	3.97±4.22[c] (0-12.96)	5.75±5.52
17	10.10±10.74 (0-35.33)	19.23±9.14 (0-32.48)	17.21±15.61 (0-47.18)	17.69±12.49
18	2.64±3.31 (0-11.8)	11.75±8.68 (0-29.9)	6.94±4.91[b] (0-17.35)	8.20±7.01
19	2.95±5.56 (0-18.14)	13.34±11.06 (0-29.12)	12.63±11.7[a] (0-33.93)	11.6±10.72
20	2.69±7.45 (0-26.97)	9.09±9.8 (0-31.79)	7.28±9.12 (0-31)	7.68±9.05
**Total**	**2.55±5.32 (0-35.33)**	**12.48±9.42 (0-44.75)**	**8.43±9.29[c] (0-47.18)**	**8.87±3.72**

Letters in the same line indicate statistically significant differences between all values: a - $p \le 0.5$; b - $p \le 0.01$; c - $p \le 0.001$.

Supplementary Table S2. Fraction of C thyrocytes in three different regions of the medial part of porcine thyroid lobe and total fraction of C thyrocytes in examined cases.

Case no.	Fraction of C thyrocytes (%)			Total fraction of C thyrocytes per case (%)
	Superficial area	Central area	Intermediate area	
	Mean±SD (Range of values)	Mean±SD (Range of values)	Mean±SD (Range of values)	Mean±SD
1	2.72±2.79 (0-9.17)	8.39±4.07 (2.4-14.46)	6.64±3.48[b] (0-11.34)	5.85±4.16
2	2.87±2.29 (0-6.42)	7.45±3.13 (0-12.06)	5.15±2.85[b] (0-9.45)	5.37±3.31
3	4.81±7.46 (0-26.09)	10.96±5.64 (0-20)	10.65±7.82[a] (0-24.17)	8.77±7.44
4	3.98±6.26 (0-18.57)	9.55±4.05 (2.63-15.93)	7.35±4.4[a] (0-14)	7.27±5.4
5	2.46±2.97 (0-8.44)	12.46±5.94 (0-21.36)	9.14±4.29[c] (3.61-18.95)	8.72±6.13
6	5.07±6.56 (0-19.58)	15.08±4.3 (9.09-24.32)	9.27±5.46[c] (0-20)	9.89±6.8
7	10.51±6.79 (0-22.22)	19.04±4.9 (10.45-27.71)	13.73±7.45[b] (1.09-23.08)	14.59±7.24
8	3.71±4.8 (0-15.46)	11.3±4.34 (5.17-19.150	6.41±3[b] (2.06-11.94)	7.59±5.12
9	3.18±4.57 (0-13.33)	12.13±4.11 (5.22-19.01)	8.94±4.24[b] (3.70-16.67)	8.15±5.63
10	3.51±3.98 (0-9.34)	12.14±7.01 (2.35-29.09)	6.89±5.17[b] (0-17.65)	7.91±6.48
11	2.69±2.96 (0-8.51)	11.67±3.52 (5.55-16.16)	8.28±6.03[c] (0.83-20.34)	7.32±5.68
12	2.94±3.28 (0-10.2)	9.84±4.18 (1.64-15.79)	5.27±3.09[c] (0-10.18)	6.15±4.52
13	6.73±6.06 (0-19.86)	17.41±6.04 (4.22-26.09)	13.75±6.27)[c] (0-23.33)	12.94±7.47
14	3.52±4.46 (0-14.28)	10.11±4.06 (4.54-20)	6.87±3.08[c] (0-12.17)	6.79±4.69
15	1.98±2.89 (0-8.28)	9.39±5.63 (0-18.96)	6.76±5.42[b] (0-16.85)	6±5.63
16	3.28±5.13 (0-15.79)	12.58±3.76 (6.56-22.58)	6.76±2.8[c] (0.93-11.54)	7.78±5.51
17	8.80±7.67 (0-24.56)	18.05±4.47 (12.93-26.73)	12.37±4.77[b] (1.39-20.45)	13.14±6.86
18	4.64±4.46 (0-13.33)	11.46±5.34 (0-22.82)	9±5.69[b] (0-22.06)	8.63±5.86
19	0.80±1.85 (0-6.78)	3.60±3.5 (0-11.63)	3.48±3.13[c] (0-8.89)	2.82±3.16
20	1.96±4.17 (0-18.05)	5.21±4.99 (0-17.81)	3.48±3.55[a] (0-10)	3.91±4.43
**Total**	**3.81±5.12 (0-26.09)**	**10.79±6.15 (0-29.09)**	**7.66±5.49[c] (0-24.17)**	**7.98±2.94**

Letters in the same line indicate statistically significant differences between all values: a - $p \le 0.5$; b - $p \le 0.01$; c - $p \le 0.001$.

Supplementary Table S3. Fraction of C thyrocytes in three different regions of the medial part of equine thyroid lobe and total fraction of C thyrocytes in examined cases.

Case no.	Fraction of C thyrocytes (%)			Total fraction of C thyrocytes per case (%)
	Superficial area	Central area	Intermediate area	
	Mean±SD (Range of values)	Mean±SD (Range of values)	Mean±SD (Range of values)	Mean±SD
1	0.72±1.62 (0-5.49)	6.12±5.14 (0-15.23)	6.45±4.93[c] (0-16.91)	4.23±4.9
2	3.87±5.21 (0-15.73)	15.00±8.29 (0-25.66)	9.02±7.06[b] (0-22.01)	8.66±8.21
3	0.12±0.46 (0-1.72)	6.37±8.06 (0-20.45)	7.66±9.46[a] (0-27.84)	4.5±7.75
4	4.77±6.79 (0-27.69)	16.91±9.34 (0-32.78)	17.17±9.98[c] (0-39.34)	12.72±10.47
5	1.42±4.35 (0-20.49)	9.33±6.65 (0-25.47)	10.08±6.17[c] (0-25.79)	6.76±6.96
6	0.34±1.43 (0-7.33)	6.52±6.76 (0-29.05)	3.51±3.9[c] (0-12.21)	3.58±5.19
7	0.67±1.8 (0-7.36)	7.94±7.13 (0-25.9)	6.86±8.33[c] (0-28.69)	4.63±7.11
8	2.46±3.93 (0-15.62)	12.29±5.64 (2.21-24.31)	8.40±7.22[c] (0-23.56)	7.54±6.99
9	0.76±2.53 (0-12.62)	8.67±7.05 (0-25.81)	5.58±5.98[c] (0-20)	4.61±6.37
10	0.43±1.56 (0-7.63)	3.24±2.43 (0-9.66)	2.74±2.5[c] (0-9.24)	2.26±2.5
11	0.77±1.96 (0-8.26)	5.85±5.06 (0-21.59)	3.32±3.44[c] (0-12.62)	3.26±4.22
12	1.53±2.51 (0-10.11)	8.20±4.75 (0-18.56)	6.65±5.46[c] (0-18.93)	5.51±5.23
13	0.60±1.76 (0-8.26)	4.54±4.15 (0-21.59)	3.03±2.99[c] (0-12.62)	2.73±3.51
14	0.78±3.13 (0-15.62)	10.89±9.38 (0-9.38)	8.70±8.68[c] (0-25.31)	6.04±8.69
15	1.35±3.64 (0-13.33)	9.98±9.11 (0-30.23)	9.24±10.6[c] (0-36.78)	6.29±9.13
16	2.03±3.18 (0-15.11)	8.89±4.88 (0-20.95)	6.53±6.12[c] (0-22.3)	5.84±5.6
17	2.57±6.06 (0-22.22)	13.68±9.64 (0-33.02)	8.66±8.37[c] (0-28.45)	8.33±9.27
18	4.40±5.79 (0-16.95)	12.61±10.89 (0-33.67)	14.38±12.13[c] (0-42.46)	9.30±10.79
19	0.79±2.68 (0-12.42)	7.90±7.66 (0-27.34)	10.21±8.75[c] (0-24.69)	6.28±7.91
20	1.77±2.77 (0-7.84)	5.53±3.25 (0-9.95)	3.58±2.63[a] (0-7.22)	3.61±3.22
**Total**	**1.55±3.73 (0-27.69)**	**8.87±7.78 (0-33.67)**	**7.44±8[c] (0-42.46)**	**5.83±2.56**

Letters in the same line indicate statistically significant differences between all values: a - p≤0.5; b - p≤0.01; c - p≤0.001.

Supplementary Table S4. Fraction of C thyrocytes in three different regions of the medial part of bovine thyroid lobe and total fraction of C thyrocytes in examined cases.

Case no.	Fraction of C thyrocytes (%)			Total fraction of C thyrocytes per case (%)
	**Superficial area**	**Central area**	**Intermediate area**	
	**Mean±SD (Range of values)**	**Mean±SD (Range of values)**	**Mean±SD (Range of values)**	**Mean±SD**
1	1.56±3 (0-10.26)	11.94±5.91 (0-19.8)	8.61±6.32[c] (0-20.89)	7.15±6.81
2	3.73±4.94 (0-18.45)	14.81±7.14 (0-23.73)	13.70±5.82[c] (0-25.49)	10.04±7.79
3	5.48±7.27 (0-26.13)	14.05±8.25 (0-25)	14.24±6.59[c] (0-24.49)	10.97±8.39
4	1.33±2.17 (0-8.84)	16.18±7.44 (0-24.72)	13.61±8.38[c] (0-32.17)	9.93±9.21
5	1.56±4.34 (0-20.26)	14.75±9.64 (0-27.47)	12.10±8.99[c] (0-28.19)	8.63±9.77
6	2.58±3.01 (0-11.16)	10.19±5.06 (1.51-22.84)	9.01±4.97[c] (0.51-20.22)	7.18±5.53
7	2.68±4.54 (0-17.24)	9.95±7.26 (0-30.61)	10.71±6.55[c] (0-24.12)	7.15±7.15
8	1.66±3.88 (0-18.44)	12.47±5.41 (2.35-23.65)	8.61±4.75[c] (0-15.58)	7.37±6.48
9	1.23±1.6 (0-5.64)	8.10±5.76 (0-22.1)	5.82±3.64[c] (0-12.8)	4.71±4.92
10	2.14±4.66 (0-18.78)	12.02±9.01 (0-26.44)	10.26±8.09[c] (0-23.98)	9.23±8.61
11	1.79±3.81 (0-17.09)	12.04±6.83 (0-26.36)	10.30±7.04[c] (0-28.39)	7.66±7.52
12	4.28±7.07 (0-26.67)	14.33±12.62 (0-32.57)	12.65±11.13[b] (0-34.74)	9.28±11.34
13	0.38±0.58 (0-1.82)	10.96±8.46 (0-21.69)	4.25±4.37[c] (0-11.84)	4.75±6.96
14	3.90±5.02 (0-13.04)	11.44±6.31 (0-21.78)	8.12±6.25[a] (0-19.12)	7.61±6.54
15	3.95±4.09 (0-11.58)	19.43±4.46 (12.59-26.36)	13.05±6.41[c] (1-21.55)	11.51±8.12
16	2.61±4.95 (0-17.16)	9.46±6.04 (0-23.56)	11.48±7.2[b] (0-25)	7.30±7.11
17	4.38±4.38 (0-12.43)	15.94±3.76 (11.32-26.31)	12.15±5.96[c] (2.63-24.21)	10.55±6.75
18	2.10±4.38 (0-14.75)	15.05±3.36 (9.28-24.56)	10.63±6.43[c] (0.78-20.89)	8.63±7.24
19	4.03±5.35 (0-18.42)	12.36±7.39 (0-25.19)	10.84±4.48[b] (0.73-16.57)	9.06±6.8
20	3.55±6.5 (0-18.18)	20.90±5.03 (12.57-28.57)	14.29±7.83[c] (0-23.39)	12.87±9.65
**Total**	**2.62±4.7 (0-26.67)**	**12.96±7.97 (0-32.57)**	**10.75±7.43[c] (0-34.74)**	**8.58±2.08**

Letters in the same line indicate statistically significant differences between all values: a - $p \leq 0.5$; b - $p \leq 0.01$; c - $p \leq 0.001$.
